# Relative Enhancement in Gadoxetate Disodium-Enhanced Liver MRI as an Imaging Biomarker in the Diagnosis of Non-Alcoholic Fatty Liver Disease in Pediatric Obesity

**DOI:** 10.3390/nu15030558

**Published:** 2023-01-20

**Authors:** Azadeh Hojreh, Julia Lischka, Dietmar Tamandl, Dariga Ramazanova, Amra Mulabdic, Susanne Greber-Platzer, Ahmed Ba-Ssalamah

**Affiliations:** 1 Department of Biomedical Imaging and Image-Guided Therapy, Medical University of Vienna, Waehringer Guertel 18-20, 1090 Vienna, Austria; 2 Clinical Division of Pediatric Pulmonology, Allergology and Endocrinology, Department of Pediatrics and Adolescent Medicine, Medical University of Vienna, Waehringer Guertel 18-20, 1090 Vienna, Austria; 3 Section for Medical Statistics, Center for Medical Statistics, Informatics and Intelligent Systems, Medical University of Vienna, Spitalgasse 23, 1090 Vienna, Austria

**Keywords:** magnetic resonance imaging, hepatic fat fraction, relative enhancement, gadolinium ethoxybenzyl DTPA, pediatric obesity, non-alcoholic fatty liver disease

## Abstract

Relative enhancement (RE) in gadoxetate disodium (Gd-EOB-DTPA)-enhanced MRI is a reliable, non-invasive method for the evaluation and differentiation between simple steatosis and non-alcoholic steatohepatitis in adults. This study evaluated the diagnostic accuracy of RE in Gd-EOB-DTPA-enhanced liver MRI and hepatic fat fraction (HFF) in unenhanced liver MRI and ultrasound (US) for non-alcoholic fatty liver disease (NAFLD) screening in pediatric obesity. Seventy-four liver US and MRIs from 68 pediatric patients (13.07 ± 2.95 years) with obesity (BMI > BMI-for-age + 2SD) were reviewed with regard to imaging biomarkers (liver size, volume, echogenicity, HFF, and RE in Gd-EOB-DTPA-enhanced MRIs, and spleen size), blood biomarkers, and BMI. The agreement between the steatosis grade, according to HFF in MRI and the echogenicity in US, was moderate. Alanine aminotransferase correlated better with the imaging biomarkers in MRI than with those in US. BMI correlated better with liver size and volume on MRI than in US. In patients with RE < 1, blood biomarkers correlated better with RE than those in the whole sample, with a significant association between gamma-glutamyltransferase and RE (*p* = 0.033). In conclusion, the relative enhancement and hepatic fat fraction can be considered as non-invasive tools for the screening and follow-up of NAFLD in pediatric obesity, superior to echogenicity on ultrasound.

## 1. Introduction

Childhood obesity has become a major health issue worldwide, associated with several co-morbidities including non-alcoholic fatty liver disease (NAFLD) [1,2]. NAFLD is defined by a fat content exceeding 5% of the liver weight, as assessed by liver biopsy, in the absence of excessive alcohol intake or other causes of steatosis [1,3]. NAFLD includes simple fat deposition (hepatic steatosis), inflammation in the liver (steatohepatitis) as well as hepatic fibrosis and cirrhosis [1]. Approximately 25% of children with NAFLD are diagnosed with non-alcoholic steatohepatitis (NASH), with the risk of developing hepatic fibrosis, which is associated with progression to cirrhosis, and with the potential to develop into hepatocellular carcinoma [4,5,6,7,8]. Cirrhosis due to NASH has been reported in children as young as 10 years of age [6]. Decompensated end-stage liver disease can also occur in children, leading to liver transplantation in early adulthood, and children with NAFLD appear to have a 13.8% higher risk of death and liver transplantation than the age- and sex-matched controls [9]. Signs of NAFLD include obesity, hepatomegaly [10], elevated transferases, or hepatic steatosis on imaging [1,11].

Evidence of steatosis in >5% of hepatocytes is required for a diagnosis of NAFLD in children and adults [12]; therefore, liver biopsy is still the gold standard, as it is the only test that provides detailed information about the hepatic architecture, degree of inflammation, and fibrosis [1]. Nevertheless, liver biopsy is an invasive procedure with potential complications [13].

Ultrasound (US) has been used as an imaging method to screen for the diagnosis of pediatric NAFLD [1,14]. It is non-invasive, easily accessible, and inexpensive, and can be used to estimate the liver size and fat deposition in the liver parenchyma, defined as an increase in echogenicity [15,16]. However, ultrasound has limitations in the diagnosis of steatosis hepatis. The hyperechogenicity of the liver parenchyma is influenced by body habitus, which affects liver echogenicity and may cause a false-positive diagnosis of fat deposition [17]. In addition, co-existing liver conditions such as fibrosis and inflammation also affect liver echogenicity [17]. US results, depending on the operator and the device, also lack reproducibility.

Magnetic resonance imaging (MRI) is operator-independent, non-invasive, and offers different methods for the quantification of fat content using chemical-shift imaging techniques [15,18,19,20]. Gadoxetate disodium (Gd-EOB-DTPA)-enhanced MRI is useful for the evaluation of chronic liver diseases, the staging of hepatic fibrosis, and for the acquisition of global and territorial liver function information [21,22]. This technique is also useful for the differentiation between simple steatosis and NASH in adults, providing a high sensitivity of 97%, but a low specificity of 63% [23]. However, MRI is expensive and not always widely available. Therefore, MRI should be used primarily as a screening imaging technique for clinical trials and research studies [24].

The aim of the study was to evaluate the diagnostic accuracy of relative enhancement in gadoxetate disodium-enhanced liver MRI and hepatic fat fraction in unenhanced liver MRI compared to ultrasound (US) as imaging biomarkers for the screening and follow-up of non-alcoholic fatty liver disease (NAFLD) in pediatric obesity compared to the clinical and metabolic characteristics.

## 2. Materials and Methods

### 2.1. Patients

All pediatric patients (younger than 18 years of age) at the Outpatient Clinic for Pediatric Obesity and Dyslipidemia of the Department of Pediatrics and Adolescent Medicine, with a body mass index (BMI) > BMI-for-age + 2 standard deviations (SD), according to the WHO obesity definition [25,26], and who underwent a liver US and a liver MRI in the Department of Biomedical Imaging and Image-guided Therapy, between January 2012 and December 2018, were retrospectively reviewed by a pediatric radiologist with 17 years of experience. Liver and spleen size and hepatic steatosis grade, estimated on US and on MR images, BMI, and routine blood biomarkers (aspartate aminotransferase [ASAT], alanine aminotransferase [ALAT], gamma-glutamyltransferase [GGT], alkaline phosphatase, total bilirubin, albumin, cholinesterase, lipase, C-reactive protein [CRP], lactate dehydrogenase [LDH], and ferritin) were acquired within eight weeks before or after the US and MRI examinations.

Exclusion criteria were the absence of tests or images included in the inclusion criteria, severe motion artifacts, and non-standardized US and MRI examinations that would prevent a retrospective liver evaluation.

One hundred and one MRIs and 307 US examinations were performed during the study period in 223 pediatric patients with obesity in the Department of Biomedical Imaging and Image-guided Therapy. Fourteen patients with seven MRIs and 13 US examinations were excluded because they were older than 18 years of age on the day of the US or MRI examination. Twenty MRIs from 19 patients were excluded because their US examinations were performed in other radiology departments and were not repeated in the Department of Biomedical Imaging and Image-guided Therapy. Two hundred and twenty US examinations were excluded because no corresponding MRIs were available, as the patients were frequently followed-up with US according to the overall management algorithm for children with suspected NAFLD [14].

**Figure 1:**
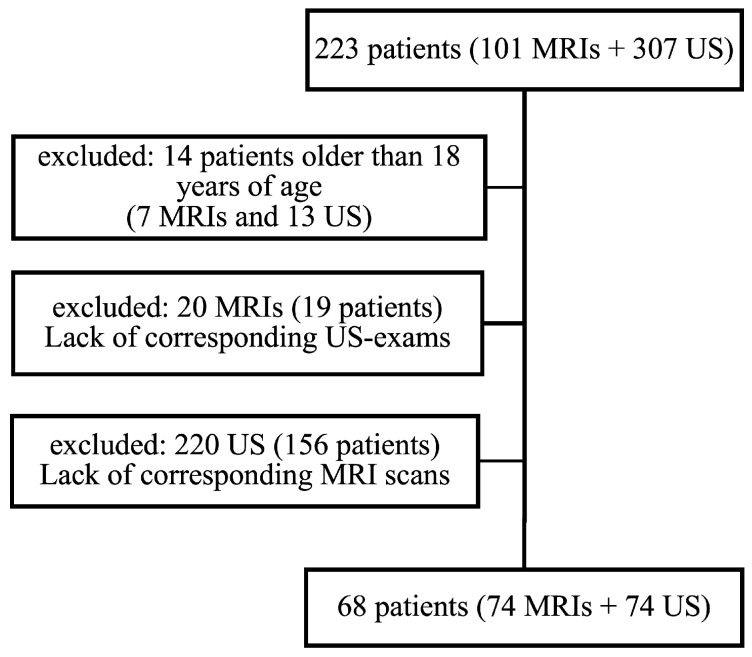
The selection process for the study patients.

A total of 68 patients met the inclusion criteria and underwent 74 US and 74 MRI examinations. The selection process is summarized in Figure 1. Table 1 presents the demographic data of the study collective.

The blood biomarkers on the examination day are presented in Supplementary Table S1. Six patients (four females and two males) were examined two times in the study period. All US examinations were unenhanced and without sedation. All but one patient had a MRI scan without sedation. This was a 1.9-year-old boy, with a homozygous leptin-receptor deficiency and a body mass index (BMI) of 51.8, who needed sedation for the MRI scan. The lowest BMI was 22.6, with a z-score of 1.77 (>BMI-for-age + 2SD), in a 9.5-year-old girl.

### 2.2. Imaging Biomarkers

The time interval between the MRI and US examinations was 0 to 145 days, but all patients had a blood sample test taken prior to both imaging examinations (US and MR-imaging) with which the imaging biomarkers were correlated.

The US examinations were performed with an ultrasound device, Toshiba Aplio400^®^ (Canon Medical Systems Corporation, Otawara, Japan), and documented according to the recommendation from the National Society for US in Medicine [27].

The MRI scans were performed on a 1.5 Tesla MR-Scanner, Siemens Magnetom Aera^®^ (Siemens Healthineers, Siemens Healthcare GmbH, Erlangen, Germany). The liver MRI examination protocol is described in Supplementary Table S2. The contrast agent was administered according to the clinical indication [22,23,28] and after dedicated, informed consent was obtained from the referring physician and the legal guardian. Gd-EOB-DTPA was applied at a dose of 0.1 mL/kg (0.025 mmol/kg) body weight, administered manually as an intravenous bolus injection. There were no adverse reactions after the intravenous application of Gd-EOB-DTPA, after either single or repeated Gd-EOB-DTPA-enhanced MR scans.

The liver size was measured (cm) on the US and MR images in three standardized lines: the anterior axillary line (AAL), the medio-clavicular line (MCL), and the lateral-sternal line (LSL). The spleen size was measured (cm) in the long axis of the spleen. The liver volume was measured on MR images with the syngo.via^®^ volumetric tool (Siemens Healthineers, Siemens Healthcare GmbH, Erlangen, Germany).

The hepatic steatosis grade was estimated in the US images depending on the echogenicity appearance of the liver parenchyma using a visual grading scale: “0” for normal echogenicity or no steatosis hepatis; “1” for mild hyperechogenicity or mild steatosis hepatis; “2” for moderate hyperechogenicity or moderate steatosis hepatis; and “3” for severe hyperechogenicity or severe steatosis hepatis.

The hepatic steatosis grade was estimated in the MR images according to the calculated hepatic fat fraction (HFF) [19]:

$$\mathrm{hepatic}\;\mathrm{fat}\;\mathrm{fraction}=100\;\times \;\frac{\mathrm{liver}\;\mathrm{signal}\;\mathrm{in}\;\text{In-phase}-\mathrm{liver}\;\mathrm{signal}\;\mathrm{in}\;\text{Opposed-phase}}{2\times \;\mathrm{liver}\;\mathrm{signal}\;\mathrm{in}\;in-phase}$$

where “0” for no hepatic steatosis if HFF <5%; “1” for mild hepatic steatosis if HHF = 5–14%; “2” for moderate hepatic steatosis if HFF = 15–29%; and “3” for severe hepatic steatosis if HFF >30% [19,29].

Liver function was estimated using the Gd-EOB-DTPA relative enhancement (RE) [22,30,31].

$$\mathrm{relative}\;\mathrm{enhancement}=\frac{\mathrm{liver}\;\mathrm{signal}\;20\;\min\mathrm{post}\;\mathrm{contrast}\;-\;\mathrm{liver}\;\mathrm{signal}\;\mathrm{of}\;\mathrm{pre}\;\mathrm{contrast}\;}{\mathrm{signalintensity}\;\mathrm{of}\;\mathrm{unenhanced}\;\mathrm{liver}\;}$$

We defined a cut-off when RE was less than 1 to correlate the estimated liver function with the blood biomarkers, as Wibmer et al. reported that RE (given as a percentage) was less than 100% in patients with liver failure [31]; In this way, we created a subgroup of patients who had an RE of less than 1 in the liver-contrast MRI. Supplementary Table S3 presents the US and MR-imaging biomarkers.

All patient imaging data were evaluated using a PACS (picture archiving and communication system, IMPAX EE^®^, Dedalus Healthcare, Bonn, Germany) on a diagnostic gray-scale monitor (Barco MDCG-3120, Brussels, Belgium).

### 2.3. Statistical Analysis

The statistical analysis and data visualization were performed using R, version 4.0.4 [32]. Spearman’s ρ correlation coefficients were calculated to evaluate the relationship between the blood biomarkers and the imaging biomarkers and the deviation between the imaging biomarkers in the US and MRI. Intraclass correlation coefficients (ICC) and their 95% confidence intervals were calculated to determine the agreement between the US and MRI measurements of liver size and spleen size.

In our sample, six patients were investigated twice. Therefore, the linear mixed models with patients as a random factor were conducted to evaluate the effect of HFF and biomarkers on the RE. The subgroup with an RE < 1 consisted of the patients investigated just once; thus, the influence of the same parameters on RE was studied using simple linear regressions. *p*-values less than 0.05 were considered significant.

### 2.4. Ethics

The study was approved by the ethics committee of the Medical University of Vienna (IRB No. 1512/2019). All procedures performed in the study that involved human participants were in accordance with the ethical standards of the institutional review board and with the 1964 Declaration of Helsinki and its later amendments. Patient consent was waived due to the retrospective data analysis.

## 3. Results

The intraclass correlation coefficients (ICC) for liver size measured in the US and MR images presented a moderate agreement for the right liver lobe (AAL: ICC = 0.620, [95%CI: 0.462–0.742]; MCL: ICC = 0.576, [95%CI: 0.407–0.709]), but a poor agreement for the left liver lobe (LSL: ICC = 0.272, [95%CI: 0.062–0.465]). For spleen size measured in the US and MR images, a good agreement was observed (ICC = 0.790, [95%CI: 0.688–0.863]).

The frequencies of the hepatic steatosis grade estimated in the US and MR images are juxtaposed in Table 2. 

Of the 30 patients with no steatosis hepatis according to the calculated HFF in the MR images, there were only 12 patients estimated to have no steatosis hepatis according to the echogenicity of the liver parenchyma, but seven with mild and 11 with moderate hyperechogenicity of the liver parenchyma. In patients with estimated mild steatosis hepatis according to the echogenicity of the liver parenchyma in ultrasound, the calculated HFF varied between no to moderate steatosis hepatis and in patients with estimated moderate steatosis hepatis in ultrasound, the calculated HFF varied between no to severe steatosis hepatis. However, if no steatosis hepatis was estimated in the ultrasound, the calculated HFF on MR images also revealed no steatosis hepatis, and an estimated severe steatosis hepatis on ultrasound varied only between moderate and severe HFF levels in the MR images.

The interclass correlation coefficient demonstrated a moderate agreement of the hepatic steatosis grade based on the HFF in MRI and visual grading of the echogenicity in US (ICC = 0.646, [95%CI:0.496–0.761]).

Table 3 presents the Spearman’s (ρ) correlation coefficients between the blood biomarkers, BMI and BMI z-score, and spleen size, with a liver size measured in the US and MR images.

In this dataset, the correlation of liver size with the blood biomarkers was generally higher for MRI than for US. The AAL measurements in the MRI correlated with total bilirubin, cholinesterase, alkaline phosphatase, ASAT, ALAT, GGT, and ferritin more strongly than the AAL measurements in the US, whereas albumin indicated a stronger correlation with the AAL measurements in the US than with those in the MRI. The correlation between BMI and liver size in the MR images was notably higher than in the US images, except for measurements in LSL, and the correlation between BMI and liver volume was the strongest. Both the liver size in the US and MRI images correlated less with the BMI-z-score than with the BMI. The correlation between liver and spleen size in the MRI was strongly higher than in the US images (Table 3).

Spleen size was also separately correlated with the blood biomarkers, BMI, and BMI z-score. The spleen size in the MR images correlated with cholinesterase and lactate dehydrogenase better than in the US images and the correlation coefficient between CRP and the spleen size was higher in the US images than in the MRI. The correlation coefficient between spleen size and BMI in the MRI and US images was approximately the same (Table 3).

Of the 58 Gd-EOB-DTPA-enhanced MR scans, there were 19 MR scans with a Gd-EOB-DTPA relative enhancement lower than 1 (median value of RE: 1.12, range 0.36–1.8 vs. median value of RE: 0.92, range 0.36–0.98) (Supplementary Table S3).

Table 4 presents the Spearman’s (ρ) correlation coefficients between the blood biomarkers, spleen size, and the grade of hepatic steatosis in the US and MR images for the whole study collective as well as for the subgroup with an RE < 1.

Across the whole study collective, the estimated hepatic steatosis grade by echogenicity in the US images correlated with albumin, cholinesterase, liver transferases, ferritin, lipase, and lactate dehydrogenase. The estimated hepatic steatosis grade by hepatic fat fraction in the MRI correlated with albumin, cholinesterase, alkaline phosphatase, liver transferases, ferritin, lipase, and lactate dehydrogenase. The correlation coefficient between the hepatic fat fraction and ALAT was higher than that with all other blood biomarkers. The relative enhancement correlated more strongly with albumin and lipase than with all the other blood biomarkers. In the subgroup with an RE < 1, the correlation of relative enhancement with blood biomarkers was higher than in the whole study collective, except for albumin. In this same subgroup, the correlation of HFF with blood biomarkers was also higher than in the whole study collective, except for GGT and albumin (Table 4).

The correlation coefficient between liver imaging biomarkers and the spleen size was weak for US as well as for MRI, either in the whole study collective or in the subgroup with RE < 1 (Table 4).

The linear mixed models showed that HFF, spleen size, and blood biomarkers had no significant influence on the Gd-EOB-DTPA relative enhancement in the whole study collective, except for albumin (*p* = 0.045), but in the subgroup with an RE < 1, GGT had a significant association with relative enhancement (*p* = 0.033) (Table 5).

**Figure 2:**
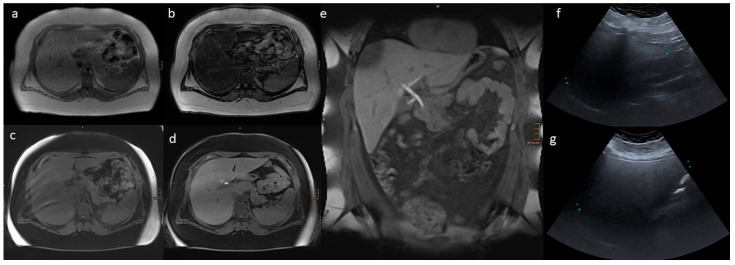
Liver MR and ultrasound images of a 14.6-year-old boy with a body mass index of 56.3 kg/m^2^: (**a**) In-phase; (**b**) opposed phase; (**c**) non-contrast T1 VIBE fat saturated; (**d**,**e**) transitional phase of Gd-EOB-DTPA-enhanced T1 VIBE fat saturated sequences; and (**f**) AAL and (**g**) MCL liver ultrasound images. In the ultrasound, a severe steatosis hepatis was estimated according to the echogenicity of the liver parenchyma. In the MRI, the liver volume was 3401 mL, and the hepatic fat fraction was 38.4% based on severe steatosis hepatis, and 0.36 was the relative enhancement.

**Figure 3:**
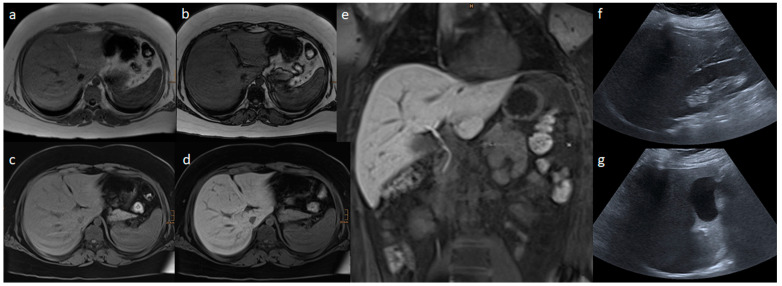
Liver MR and ultrasound images of a 13.7-year-old boy with a body mass index of 40.1 kg/m^2^: (**a**) In-phase; (**b**) opposed phase; (**c**) non-contrast T1 VIBE fat saturated; (**d**,**e**) transitional phase of Gd-EOB-DTPA-enhanced T1 VIBE fat saturated sequences; (**f**) AAL; and (**g**) MCL liver ultrasound images. In the ultrasound, a mild steatosis hepatis was estimated based on the echogenicity of the liver parenchyma. In the MRI, the liver volume was 1953 mL, and the hepatic fat fraction was 15.9% based on moderate steatosis hepatis, and 1.3 was the relative enhancement.

Figure 2 and Figure 3 present the MRI and ultrasound images of two patients in the study collective.

## 4. Discussion

We could demonstrate, in our collective, that MR-imaging biomarkers are more accurate for the screening and follow-up of NAFLD in pediatric obesity than US-imaging biomarkers.

In the study population, although the agreement of the liver size measurements in the US and MR images was moderate, the correlation of an increased liver size with the routine blood biomarkers was higher for MRI than for US. The correlation of an increased liver size with a pathologic BMI was higher for MRI than for US and seemed to be the strongest correlation.

While in our study collective a moderate agreement was observed between the grade of hepatic steatosis according to HFF and visual grading of liver echogenicity, it was noticeable that if the echogenicity of the liver parenchyma in the US images was normal and no steatosis hepatis was diagnosed, there was also no steatosis hepatis estimated according to the calculated HFF. In the cases with severe steatosis hepatis based on severe hyperechogenicity of the liver parenchyma in the US images, the calculated HFF varied only in a range from moderate to severe steatosis hepatis. However, the calculated HFF in the cases with a mild steatosis hepatis in the US images varied between no and moderate steatosis hepatis. In patients with a moderate steatosis hepatis on ultrasound, the calculated HFF varied between a wide range of steatosis hepatis levels, from no to severe steatosis hepatis. Obviously, the grade of fatty liver could not be correctly assigned based on the mild to moderate hyperechogenicity of the liver parenchyma. Pacifico et al. also observed that if ultrasound showed a moderate to severe steatosis in children with NAFLD, MRI presented a wide range of HFF within both categories of ultrasound steatosis severity [33].

The European Society for Pediatric Gastroenterology, Hepatology and Nutrition Hepatology Committee included US in the overall management algorithm for children with suspected NAFLD [14]. For clinical purposes, currently, the diagnosis of NAFLD is usually based on US imaging of liver echogenicity, and eventually, increased liver transferase activity and ALAT, in combination with liver US, as an indicator of NAFLD [14]. However, hepatic steatosis mostly appears in US as a diffuse increase in echogenicity due to the increased parenchymal reflectivity caused by the intracellular accumulation of fat inclusions [15,16]. The hyperechogenicity of the liver parenchyma is influenced by body habitus (e.g., in adipose patients) because abdominal fat may attenuate the US beam, which affects liver echogenicity and may cause a false-positive diagnosis of fat deposition [17]. Co-existing liver conditions such as fibrosis and inflammation also affect liver echogenicity [17]. US results, depending on the operator and the device, lack reproducibility, while MRI is operator-independent and enables quantitative measurement of the lipid content in the liver using chemical-shift imaging techniques [18,19], and can monitor the progression or regression of hepatic steatosis in patients, if children with obesity lose weight [33,34].

The European Association for the Study of the Liver guidelines highlight the role of MRI, primarily as a screening imaging technique for clinical trials and research studies [20,24].

In our study collective, however, the correlation between HFF and liver transferase was higher than that for the visual grading of liver echogenicity. The correlation coefficient between HFF and ALAT was also higher than that for all other blood biomarkers (whole study group: ρ = 0.628 vs. subgroup with an RE < 1: ρ = 0.756) compared to the correlation coefficient between echogenicity, as graded visually, for steatosis hepatis and ALAT (ρ = 0.543).

In the subgroup (RE < 1), the RE correlated with the blood biomarkers more strongly than in the whole study sample, except for albumin. The correlation of HFF (measured values) with blood biomarkers, except for GGT and albumin, was also higher. The univariate linear mixed models showed that neither the imaging biomarkers (liver size, HFF, and spleen size) nor the blood biomarkers had a significant influence on RE in the whole collective. Only albumin had a slightly significant influence on RE (*p* = 0.045). In the subgroup (RE < 1), GGT had a significant influence on the relative enhancement (*p* = 0.033). However, the significant influence of GGT on RE in the subgroup (RE < 1) was stronger than the influence of albumin on the RE in the whole study sample.

Kukuk et al. reported that relative enhancement using Gd-EOB-DTPA correlated with routinely used liver function tests, and that hepatobiliary MRI served as a valuable biomarker of liver function in patients with liver diseases [28]. Bastati et al. reported that Gd-EOB-DTPA relative enhancement was significantly lower in patients with NASH than in patients with simple steatosis in adulthood [23].

Epidemiological studies have indicated an association between an elevated GGT activity level and the risk of coronary heart disease or coronary heart disease-related mortality [35]. GGT has also been suggested as a potentially reliable and non-invasive biomarker for the estimation of cardiovascular risk in pediatric obesity and NAFLD [36].

Interestingly, HFF showed no significant influence on RE in either the whole study collective or in the subgroup (RE < 1), even though the *p*-value for HFF influence on RE was lower in the subgroup (RE < 1) (*p* = 0.059) than in the whole study collective (*p* = 0.687). We surmise that this association could be attributable to the incipient development of NASH. There have been reports of increased HFF in NASH more often than in simple steatosis [37].

Liver biopsy is the gold standard with which to provide detailed information about the hepatic architecture, degree of inflammation, and fibrosis [1], and to exclude other treatable diseases and assess advanced clinical disease prior to pharmacological or surgical interventions [14]. Nevertheless, liver biopsy is an invasive procedure with potential complications including bleeding, infection, and death [13]. It is also subject to sampling error and inter-observer variability [1]. However, these potential life-threatening complications of liver biopsy cannot be justified in light of the long-term mortality of children with obesity. Therefore, it is not a feasible option for the diagnosis and follow-up of NAFLD in pediatric obesity.

Based on our study results, it can be hypothesized that a decrease in RE of less than 1 could be attributed to NASH, as also presented by Bastati et al. [23], and only in this subgroup could liver biopsy possibly be justified.

### Study Limitations

1. Due to the small sample size, the results should be interpreted with caution. Because of the multiple tests, the statistics dictate a Bonferroni correction for univariate linear regression analyses, with a *p*-value less than 0.0045 considered significant. Under this consideration, no results can be interpreted as significant due to the small sample size. Further prospective studies with larger sample sizes could confirm the study results.
2. MRI is expensive and not always widely available, compared to ultrasound.
3. RE assessment requires gadolinium methoxybenzyl-DTPA, so this method should be reserved only for pediatric patients with a clinical suspicion of NASH prior to liver biopsy.

## 5. Conclusions

Relative enhancement and hepatic fat fraction, as imaging biomarkers, are superior to visually graded echogenicity in ultrasound, and thus could serve as non-invasive tools for the diagnosis and follow-up of non-alcoholic fatty liver disease in pediatric obesity. We recommend that the hepatic fat fraction and relative enhancement on gadoxetate disodium-enhanced liver MRI should also be included as imaging biomarkers, in addition to ultrasound in the management of NAFLD in pediatric obesity, particularly in patients with a clinical suspicion of NASH prior to liver biopsy.

## Figures and Tables

**Table 1:** Demographics.

Characteristics	Value (%)	Mean (±SD)	Median	Range
No. Patients	68			
Females (%)	24 (35.3)			
Age (all patients, years)		13.07 (2.95)	13.2	1.9–17.8
Body mass index (all patients)		34.09 (7.16)	32.15	22.6–56.3
Body mass index z-score (all patients)		2.74 (0.68)	2.68	1.70–6.20
No of US examinations	74			
No of MRI scans	74			
Gd-EOB-DTPA ^a^-enhanced MRI scans (%)	58 (78.4)			
Unenhanced MRI scans (%)	16 (21.6)			
MRI scans under sedation (%)	1 (1.35)			
Time interval between US and MRI (days)		24 (33.35)	10	0–145
Time interval between US and blood sampling (days)		0	0	0
Time interval between MRI and blood sampling (days)		0	0	0

^a^ Gd-EOB-DTPA = gadoxetate disodium.

**Table 2:** Juxtaposition of patients based on echogenicity and hepatic fat fraction levels.

			Hepatic Fat Fraction Levels			
			no	mild	moderate	severe
		Value (%)	30 (40.54%)	12 (16.22%)	20 (27.02%)	12 (16.22%)
**Echogenicity levels**	No	12 (16.22%)	12	0	0	0
	Mild	15 (20.27%)	7	4	4	0
	Moderate	32 (43.24%)	11	8	8	5
	Severe	15 (20.27%)	0	0	8	7

**Table 3:** Spearman’s (ρ) correlation coefficients between blood biomarkers, BMI ^h^, and BMI z-score with liver and spleen size measured in the US and MR images.

	Liver Size on US Images			Spleen Size on US Images	Liver Size on MR Images				Spleen Size on MR Images
	AAL ^a^ in cm	MCL ^b^ in cm	LSL ^c^ in cm	Long Axis of the Spleen in cm	AAL in cm	MCL in cm	LSL in cm	Liver Volume in mL	Long Axis of the Spleen in cm
Total bilirubin	0.067	0.147	0.023	−0.037	0.215	0.264	0.129	0.214	−0.042
Albumin	0.328	0.153	0.001	−0.145	0.175	0.089	0.242	0.185	0.055
Cholinesterase	−0.001	0.249	0.262	0.039	0.219	0.111	0.370	0.414	0.250
Alkaline phosphatase	−0.137	−0.004	−0.058	−0.135	−0.209	−0.261	−0.107	−0.201	−0.112
ASAT ^d^	0.221	0.290	0.187	−0.220	0.283	0.104	0.208	0.243	−0.104
ALAT ^e^	0.257	0.362	0.249	0.193	0.341	0.247	0.338	0.392	0.017
GGT ^f^	0.239	0.333	0.287	0.003	0.335	0.107	0.258	0.430	0.090
Ferritin	0.068	0.151	0.090	−0.097	0.222	0.111	0.119	0.192	−0.041
Lipase	0.045	0.154	−0.110	−0.016	0.002	−0.071	0.129	0.010	−0.103
LDH ^g^	0.154	0.188	−0.004	−0.288	0.022	−0.051	−0.092	−0.046	−0.311
BMI ^h^	0.162	0.247	0.283	0.363	0.276	0.447	0.154	0.481	0.365
BMI z-score	−0.070	0.044	0.092	0.203	−0.007	0.196	−0.062	0.183	0.151
CRP ^i^	0.094	−0.033	0.087	0.360	0.090	0.166	−0.187	0.029	0.043
Spleen size * (on US or MRI, respectively)	0.261	0.347	0.205	1	0.534	0.557	0.553	0.604	1

^a^ AAL = anterior axillary line, ^b^ MCL = medio-clavicular line, ^c^ LSL = lateral-sternal line, ^d^ ASAT = aspartate aminotransferase, ^e^ ALAT = alanine aminotransferase, ^f^ GGT = gamma-glutamyltransferase, ^g^ LDH = lactate dehydrogenase, ^h^ BMI = body mass index, ^i^ CRP = C-reactive protein. * Spleen size was measured in the long axis of the spleen.

**Table 4:** Spearman’s (ρ) correlation coefficients between the blood biomarkers and spleen size and estimated hepatic steatosis grade in the US and MRI and relative enhancement measured in the MRI.

	US Images of the Whole Study Sample	MR Images of the Whole Study Sample			MR Images of the Subgroup with RE ^f^ < 1		
	Hepatic Steatosis Grade Based on Echogenicity	Hepatic Steatosis Grade Based on Hepatic Fat Fraction	Hepatic Fat Fraction (Absolute Measured Value)	Relative Enhancement	Hepatic Steatosis Grade Based on Hepatic Fat Fraction	Hepatic Fat Fraction (Absolute Measured Value)	Relative Enhancement
Total bilirubin	0.007	0.079	−0.000	0.035	0.106	−0.002	−0.213
Albumin	0.280	0.249	0.251	−0.291	−0.175	−0.082	−0.106
Cholinesterase	0.517	0.367	0.353	0.071	0.465	0.461	−0.288
Alkaline phosphatase	0.057	0.190	0.216	0.008	0.337	0.381	0.066
ASAT ^a^	0.452	0.544	0.507	0.032	0.502	0.535	−0.311
ALAT ^b^	0.543	0.627	0.628	0.149	0.682	0.756	−0.261
GGT ^c^	0.412	0.368	0.421	0.054	0.128	0.210	−0.322
Ferritin	0.292	0.391	0.393	0.003	0.635	0.637	−0.058
Lipase	0.165	0.162	0.139	0.299	−0.706	−0.771	0.618
LDH ^d^	0.151	0.345	0.334	−0.105	0.452	0.418	−0.244
CRP ^e^	−0.045	0.077	0.044	0.033	−0.308	−0.309	0.103
Spleen size *	0.100	0.134	0.091	−0.052	−0.099	−0.023	−0.173

^a^ ASAT = aspartate aminotransferase, ^b^ ALAT = alanine aminotransferase, ^c^ GGT = gamma-glutamyltransferase. ^d^ LDH = lactate dehydrogenase, ^e^ CRP = C-reactive protein, ^f^ RE = relative enhancement. * Spleen size was measured in the long axis of the spleen.

**Table 5:** Mixed and simple linear regression analyses of factors * associated with relative enhancement for the whole sample and for the subgroup with an RE ^g^ < 1.

	Whole Study Sample (*n* = 58)				Sample with an RE < 1 (*n* = 19)			
	Beta	95% CI ^h^		*p*	Beta	95% CI		*p*
		LL ^i^	UL ^j^			LL	UL	
HFF ^a^	0.001	−0.004	0.007	0.687	−0.006	−0.011	−0.000	0.059
Total bilirubin	−0.005	−0.272	0.263	0.973	0.051	−0.220	0.321	0.718
Albumin	−0.028	−0.055	−0.001	0.045	−0.016	−0.057	0.025	0.446
Cholinesterase	−0.003	−0.043	0.038	0.902	−0.078	−0.163	0.008	0.100
Alkaline phosphatase	−0.000	−0.001	0.001	0.979	0.000	−0.001	0.001	0.749
ASAT ^b^	−0.000	−0.003	0.002	0.790	−0.003	−0.008	0.002	0.288
ALAT ^c^	0.000	−0.001	0.001	0.856	−0.001	−0.004	0.001	0.409
GGT ^d^	−0.000	−0.001	0.001	0.731	−0.010	−0.018	−0.002	0.033
Ferritin	0.001	−0.000	0.002	0.113	0.001	−0.002	0.003	0.677
LDH ^e^	−0.000	−0.001	0.000	0.309	−0.000	−0.001	0.001	0.813
CRP ^f^	0.040	−0.080	0.161	0.513	−0.002	−0.240	0.237	0.990
Spleen size	−0.023	−0.062	0.016	0.253	−0.031	−0.072	0.011	0.163

^a^ HFF = hepatic fat fraction, ^b^ ASAT = aspartate aminotransferase, ^c^ ALAT = alanine aminotransferase, ^d^ GGT = gamma-glutamyltransferase, ^e^ LDH = lactate dehydrogenase, ^f^ CRP = C-reactive protein. ^g^ RE = relative enhancement ^h^ CI = confidence interval, ^i^ LL = lower limit, ^j^ UL = upper limit. * Lipase was excluded due to missing values (72% and 92%, respectively).

## Data Availability

The datasets generated or analyzed during the study are included in this published article and the supplementary materials. There are four supplementary tables submitted for review.

## Supplementary Materials

The following supporting information can be downloaded at: https://www.mdpi.com/article/10.3390/nu15030558/s1, Table S1: Blood biomarkers of the study collective; Table S2: Liver MR scan protocol with Gd-EOB-DTPA; Table S3: Imaging biomarkers of the liver and spleen in the US and MR images.
