# Supplementary material for: Relative Enhancement in Gadoxetate Disodium-Enhanced Liver MRI as an Imaging Biomarker in the Diagnosis of Non-Alcoholic Fatty Liver Disease in Pediatric Obesity
Source: Nutrients. 2023 Jan 20;15(3):558. doi: 10.3390/nu15030558 (PMC9921256; doi:10.3390/nu15030558)
Supplement: Supplementary file 1 [file nutrients-15-00558-s001.zip › nutrients-2116750-supplementary.pdf]

Supplementary Materials

Table S1 Blood biomarkers of the study collective

Characteristics	Value	Mean (±SD)	Median	Range
**Blood biomarkers on US[a] examination day**				
**Total bilirubin**	73	0.54 (0.29)	0.46	0.13-1.68
**Albumin**	61	45.60 (3.29)	45.70	35.70-54.10
**Cholinesterase**	42	221.70 (1375.07)	9.76	5.77-8921.00
**Alkaline phosphatase**	73	174.62 (93.01)	166.00	44.00-492.00
**ASAT[c]**	74	42.78 (35.72)	31.00	15.00-209.00
**ALAT[d]**	74	67.23 (81.24)	37.50	16.00-536.00
**GGT[e]**	74	38.74 (72.26)	19.50	9.00-493.00
**Ferritin**	71	65.83 (72.37)	51.40	14.00-567.80
**Lipase**	20	76.90 (83.25)	60.00	18.00-403.00
**LDH[f]**	73	250.17 (78.91)	242.00	94.40-556.00
**CRP[g]**	74	0.75 (0.72)	0.61	0.00-5.05
**Blood biomarkers on MRI[b] examination day**				
**Total bilirubin**	73	0.53 (0.27)	0.46	0.20-1.68
**Albumin**	60	45.68 (3.42)	45.85	38.00-54.10
**Cholinesterase**	42	221.71 (1375.07)	9.76	5.77-8921.00
**Alkaline phosphatase**	74	176.15 (93.78)	167.50	44.00-492.00
**ASAT**	74	42.84 (34.59)	31.00	14.00-204.00
**ALAT**	74	63.47 (70.02)	36.00	15.00-398.00
**GGT**	74	37.61 (68.91)	18.50	9.00-493.00
**Ferritin**	71	67.29 (72.90)	52.70	14.00-567.80
**Lipase**	20	77.80 (83.24)	66.00	15.00-403.00
**LDH**	73	263.82 (82.43)	248.00	145.00-556.00
**CRP**	74	0.73 (0.78)	0.50	0.00-5.05

[a] US = ultrasound, [b] MRI = magnetic resonance imaging, [c] ASAT = aspartate aminotransferase, [d] ALAT = alanine aminotransferase, [e] GGT =gamma-glutamyltransferase, [f] LDH = lactate dehydrogenase, [g] CRP = C-reactive protein

Data presented are absolute numbers or mean value with standard deviation (±SD), median, and range (Min-Max), when applicable.

Table S2 Liver MR scan protocol with Gd-EOB-DTPA

**Sequences**	**Slice orientation**	**Matrix**	**Voxel mm**	**FOV mm**	**SL mm**	**gap mm**	**TR ms**	**TE ms**	**FA Degree**	**Time s**
GRE T1 (2D flash) in-phase	Axial	320x320	0.5x0.5x5	350	5	1	130	2.38	70	21
GRE T1 (2D flash) opposed-phase	Axial	320x320	0.5x0.5x5	350	5	1	130	4.85	70	21
T2 HASTE FatSat thick radial	Coronal	384x384	0.8x0.8x50	300	50	25	4500	750	180	62
MRCP sequence with MIP	Coronal	256x256	1.1x1.1x1	280	1		2000	703	140	235
T2 HASTE	Axial	320x320	1.1x1.1x5	350	5	1	1000	95	160	47
T1 VIBE FatSat non-contrast	Axial	320x320	1.1x1.1x2.5	350	2.5	0.5	4.76	2.29	10	16
T1 VIBE FatSat dynamics (arterial and portal-venous)	Axial	320x320	1.1x1.1x2.5	350	2.5	0.5	4.76	2.29	10	16x3
T1 VIBE FatSat 5 min post contrast (equilibrium)	Coronal	288x288	1.3x1.3x2.2	380	2.2	0.44	2.97	1.38	10	16
T1 VIBE FatSat 5 min post contrast (equilibrium)	Axial	320x320	1.1x1.1x2.5	350	2.5	0.5	4.76	2.29	10	16
T2 HASTE FatSat	Axial	320x320	1.1x1.1x5	350	5	1	1000	95	160	47
T2 HASTE	coronal	256x256	1.4x1.4x4.5	350	4.5	0.9	1000	102	180	45
DWI TSE-EP/ADC	Axial	192x192	1.8x1.8x5	350	5	1	5100	67		133
T2 TSE FatSat triggered	Axial	384x384	0.9x0.9x5	350	5	1	2000	105	160	260
T1 VIBE FatSat 20 min post contrast (hepatobiliary)	Axial	320x320	1.1x1.1x2.5	350	2.5	0.5	4.76	2.29	10	16
T1 VIBE FatSat 20 min post contrast (hepatobiliary)	Coronal	288x288	1.3x1.3x2.2	380	2.2	0.44	2.97	1.38	10	16

HASTE = Half-Fourier Acquisition Single-shot Turbo spin Echo imaging, DWI TSE-EP/ADC = Diffusion-Weighted Imaging Turbo Spin Echo-Echo-Planar, MRCP= Magnetic Resonance Cholangiopancreatography; MIP=maximum intensity projection; GRE = gradient echo, VIBE = Volumetric Interpolated Breath-hold Examination, FOV = field of view, Voxel = voxel size, SL = slice thickness, TR = repetition time, TE = echo time, FA = flip angle, Time = acquisition time.

Table S3 Imaging biomarkers of liver and spleen on US and on MR images

Characteristics	Value (%)	Mean (±SD)	Median	Range
**Imaging biomarkers on US**				
• **Liver size measured on US (cm)**				
- **AAL**[a]		14.65 (2.23)	14.95	9.70-19.30
- **MCL**[b]		14.22 (2.26)	14.00	6.70-19.50
- **LSL**[c]		11.19 (2.51)	11.00	5.60-17.60
• **Hyperechogenicity of liver parenchyma**				
- **No**	12 (16.22)			
- **Mild**	15 (20.27)			
- **Moderate**	32 (43.24)			
- **Severe**	15 (20.27)			
• **Spleen size* measured on US (cm)**		10.88 (1.44)	11.00	6.70-13.60
**Imaging biomarkers on MRI**				
• **Liver size measured on MRI (cm)**				
- **AAL**		17.02 (2.59)	16.55	12.50-24.50
- **MCL**		10.11 (1.84)	10.00	5.60-15.00
- **LSL**		6.96 (1.17)	6.85	4.80-12.00
• **Hepatic fat fraction**				
- **<5%**	30 (40.54)			
- **5-14%**	12 (16.22)			
- **15-29%**	20 (27.03)			
- **> 30%**	12 (16.22)			
• **Liver volume calculated by syngo.via® (ml)**		1791.14 (562.03)	1743.50	861-3401
• **Hepatic fat fraction**		13.87 (13.57)	9.60	-3-45
• **Relative enhancement of study collective**	58	1.12 (0.28)	1.10	0.36-1.80
• **RE**[d] **in subgroup with RE <1**	19	0.85 (0.18)	0.92	0.36-0.98
• **Spleen size* measured on MRI (cm)**		11.79 (1.81)	11.85	7.50-16.00

[a] AAL = anterior axillary line, [b] MCL = medio-clavicular line, [c] LSL = lateral-sternal line, [d] RE = relative enhancement.

*Spleen size was measured in the long axis of the spleen.

Data presented are absolute numbers or mean value with standard deviation (±SD), median, and range (Min-Max), when applicable. Percentages that apply to the respective groups, or to the whole cohort, are presented in parentheses.
